# Gantenerumab reduces amyloid-β plaques in patients with prodromal to moderate Alzheimer’s disease: a PET substudy interim analysis

**DOI:** 10.1186/s13195-019-0559-z

**Published:** 2019-12-12

**Authors:** Gregory Klein, Paul Delmar, Nicola Voyle, Sunita Rehal, Carsten Hofmann, Danielle Abi-Saab, Mirjana Andjelkovic, Smiljana Ristic, Guoqiao Wang, Randall Bateman, Geoffrey A. Kerchner, Monika Baudler, Paulo Fontoura, Rachelle Doody

**Affiliations:** 1 Roche Pharma Research and Early Development, Basel, Switzerland; 2 Roche/Genentech Product Development, Neuroscience, Basel, Switzerland; 3 Roche Products Ltd, Welwyn Garden City, UK; 4 Washington University School of Medicine in St. Louis, St. Louis, MO USA; 5 Genentech, Inc., South San Francisco, CA USA

**Keywords:** Alzheimer’s disease, Amyloid-β plaque, Centiloid, Disease-modification therapies, Gantenerumab, Florbetapir, Open-label extension, Positron emission tomography

## Abstract

**Background:**

We previously investigated low doses (105 or 225 mg) of gantenerumab, a fully human monoclonal antibody that binds and removes aggregated amyloid-β by Fc receptor-mediated phagocytosis, in the SCarlet RoAD (SR) and Marguerite RoAD (MR) phase 3 trials. Several lines of evidence suggested that higher doses may be necessary to achieve clinical efficacy. We therefore designed a positron emission tomography (PET) substudy to evaluate the effect of gantenerumab uptitrated to 1200 mg every 4 weeks on amyloid-β plaques as measured using florbetapir PET in patients with prodromal to moderate Alzheimer’s disease (AD).

**Methods:**

A subset of patients enrolled in the SR and MR studies who subsequently entered the open-label extensions (OLEs) were included in this substudy. Patients were aged 50 to 90 years with a clinical diagnosis of probable prodromal to moderate AD and were included based on a visual read of the original screening scan in the double-blind phase. Patients were assigned to 1 of 5 titration schedules (ranging from 2 to 10 months) with a target gantenerumab dose of 1200 mg every 4 weeks. The main endpoint of this substudy was change in amyloid-β plaque burden from OLE baseline to week 52 and week 104, assessed using florbetapir PET. Florbetapir global cortical signal was calculated using a prespecified standard uptake value ratio method converted to the Centiloid scale.

**Results:**

Sixty-seven of the 89 patients initially enrolled had ≥ 1 follow-up scan by August 15, 2018. Mean amyloid levels were reduced by 39 Centiloids by the first year and 59 Centiloids by year 2, a 3.5-times greater reduction than was seen after 2 years at 225 mg in SR. At years 1 and 2, 37% and 51% of patients, respectively, had amyloid-β plaque levels below the amyloid-β positivity threshold.

**Conclusion:**

Results from this exploratory interim analysis of the PET substudy suggest that gantenerumab doses up to 1200 mg resulted in robust amyloid-β plaque removal at 2 years. PET amyloid levels were consistent with sparse-to-no neuritic amyloid-β plaques in 51% of patients after 2 years of therapy. Amyloid reductions were similar to those observed in other placebo-controlled studies that have suggested potential clinical benefit.

**Trial registration:**

ClinicalTrials.gov, NCT01224106 (SCarlet RoAD) and NCT02051608 (Marguerite RoAD).

## Background

Approximately 50 million people live with dementia worldwide, and this number is expected to grow to 82 million by 2030 and 152 million by 2050 [1]. Alzheimer’s disease (AD) is the most common form of dementia, accounting for 60 to 70% of cases; together, AD and other types of dementia are the fifth leading cause of death worldwide [2]. Currently, there are no disease-modifying therapies that can delay disease course, prevent progression, or provide a cure [3]. Available pharmacological treatments for AD dementia offer mild symptomatic benefit with no effect on the underlying neuropathology of the disease [4–10].

AD pathophysiology is characterized by the progressive accumulation of amyloid-β plaques predominantly comprising amyloid-β peptides around neurons and the formation of intracellular neurofibrillary tangles containing pathologic tau protein [11]. Deposition of amyloid-β plaques in the brain parenchyma is likely to occur decades before clinical symptoms manifest [11, 12]. The extent to which different amyloid-β species contribute to the pathophysiology of AD remains uncertain [13], although in vitro and ex vivo evidence suggest both soluble oligomers and aggregated plaques are implicated in neurotoxic effects [13–15]. This hypothesis is being tested in multiple clinical trials evaluating drugs that can attenuate the accumulation and/or promote the removal of amyloid-β in patients with AD [16–19].

Gantenerumab is a fully human anti-amyloid-β IgG1 monoclonal antibody that binds with high affinity to aggregated amyloid-β species and removes amyloid-β plaques via Fcγ receptor-mediated microglial phagocytosis [20–22]. Gantenerumab neutralizes the neurotoxic effect of oligomeric amyloid-β_42_ in vivo [22]. The effect of low-dose subcutaneous (SC) gantenerumab on cognition and function was investigated in 2 global phase 3 studies in patients with prodromal AD (SCarlet RoAD [SR]; NCT01224106; *n* = 799) and mild AD (Marguerite RoAD [MR]; NCT02051608; *n* = 389) [23, 24]. Dosing in SR was suspended after interim futility analyses revealed a low likelihood of meeting the primary endpoint at the doses studied (105 and 225 mg SC every 4 weeks [q4w]). Recruitment for MR was subsequently stopped, although dosing continued. Overall, data gathered during the double-blind phase of SR suggest that doses studied were safe and well tolerated [23]. A dose-dependent decrease in brain amyloid-β plaque burden was observed by week 100 (measured by positron emission tomography [PET]). Furthermore, dose-dependent downstream pharmacodynamic effects were observed, including reductions in the cerebrospinal fluid biomarkers phosphorylated tau, total tau, and neurogranin [23]. A post hoc subgroup analysis of patients determined to be fast progressors in SR (according to Functional Assessment Questionnaire results, normalized hippocampal volume, and Clinical Dementia Rating Scale Sum of Boxes [CDR-SB] score at baseline [25]) also suggested a dose-dependent slowing of decline in the Alzheimer’s Disease Assessment Scale cognitive subscale (ADAS-Cog) 13, Cambridge Neuropsychological Test Automated Battery, and Mini-Mental State Examination (MMSE) after 2 years of treatment [23, 25].

These observations suggest that higher doses of gantenerumab may have clinically relevant effects on cognition and function in patients with AD, especially in the early stages of the disease [23]. Accordingly, open-label extension (OLE) phases incorporating PET substudies were initiated for SR and MR to assess the short- and long-term safety and pharmacodynamic effect of gantenerumab SC q4w uptitrated to a maximum dose of 1200 mg. Interim results of the ongoing, exploratory OLE PET substudies are reported here.

## Methods

### Study design

Complete study design and methodologic details of the MR and SR studies have been described elsewhere [23, 24]. Patients aged 50 to 90 years with a clinical diagnosis of probable prodromal or mild AD based on the National Institute of Neurological and Communicative Disorders and Stroke/Alzheimer’s Disease and Related Disorders Association criteria and a positive visual amyloid PET assessment from the original double-blind screening examinations of the MR and SR studies were eligible to enroll. Patients in SR who received double-blind treatment and had ≥ 1 follow-up visit and patients who were currently enrolled in MR were eligible for OLE participation. All patients entering the OLE at centers already involved in the SR and MR PET substudies of brain amyloid-β imaging were eligible for the OLE PET substudy.

All SR and MR patients (including those previously on placebo) received subcutaneous gantenerumab in the OLE, which was uptitrated to a maximum dose of 1200 mg each month (Additional file 3: Figure S1). The dose-titration scheme used was based on patients’ apolipoprotein E (*APOE*) genotype (*APOEε4* carrier vs non-carrier) and the last double-blind treatment dose (gantenerumab 225 mg, gantenerumab 105 mg, or placebo). Gradual uptitration schemes were used to reach the target dose of 1200 mg while decreasing the risk of adverse events, particularly amyloid-related imaging abnormalities (ARIA) representative of vasogenic edema (ARIA-E). The target dose was reached within 6 to 10 months in SR OLE patients and 2 to 6 months in MR OLE patients.

The work described was carried out in accordance with the Declaration of Helsinki. Written informed consent was provided by patients as deemed applicable by institutional review boards and/or independent ethics committees.

### Patient subgrouping and data cutoff

At the start of the OLE studies, there were considerable differences between SR and MR patients with respect to prior exposure and stage of AD. Therefore, for the purposes of this analysis, patients were divided into three groups. Patients in MR were divided into those who received active drug during the double-blind phase (MR double-blind active [MR-DBA]) and those who received placebo (MR double-blind placebo [MR-DBP]), while patients in SR were pooled together in a single group, regardless of assigned treatment in the double-blind phase, as they were all off treatment for 16 to 19 months prior to OLE higher dosing. The analyses presented here are based on a clinical data date of August 15, 2018.

### Safety and clinical treatment response monitoring

Patients were monitored by one external independent monitoring committee until most patients reached the target dose, after which monitoring was continued by an internal monitoring committee. Disease severity and cognitive measures included the MMSE, CDR-SB, and ADAS-Cog 11. Safety was evaluated by adverse event reporting, blood safety testing, vital sign assessments, physical and neurological examinations, electrocardiography, and brain magnetic resonance imaging (MRI). Prior gantenerumab studies have shown that the most common safety-related events were ARIA and injection-site erythema [23]. Interim safety results for the full SR and MR OLE populations have been reported separately and indicate that no new safety signals were identified compared with lower dosing levels [24, 26]. This work will report relations found between amyloid PET data and both ARIA and clinical treatment response in patients included in the PET substudy.

### Amyloid-β plaque PET imaging

Amyloid PET scans were scheduled at baseline (defined as prior to OLE day 1 dosing), week 52, and week 104 of OLE treatment. To minimize patient burden, a new OLE baseline PET scan was not required if the patient had received one during the double-blind phase within 9 to 12 months prior to OLE dosing. Therefore, for many patients, the duration between OLE baseline and week 52 is considerably longer than 52 weeks.

All PET data were obtained using a target dose of 370 (± 10%) Becquerel (MBq) ^18^F-florbetapir injected intravenously. A 15-min scan was obtained starting at 50 min after ^18^F-florbetapir injection. PET data were checked for quality and uniformly preprocessed; a global cortical signal was then calculated using a prespecified standard uptake value ratio (SUVR) method identical to the method reported for analysis in the SR double-blind study [21]. Briefly, that method computed the volume-weighted, gray matter-masked SUVR of six bilateral cortical regions from the Automated Anatomical Labelling (AAL) template, normalized by a cerebellar cortex reference region [27, 28]. The original screening 3D T1-weighted MRI was segmented in the subject’s space, then aligned to the AAL template space to perform the gray-matter masking.

To gain further insight and to allow better comparison of results from other studies, SUVR values were converted to Centiloid values. The Centiloid framework has the useful property of rescaling SUVR values obtained via different reference regions, methodologies, or even tracers to a common scale anchored by Centiloid values of 0 and 100, which correspond to the transformed SUVR mean values of a young control group and AD group, respectively [29]. Briefly, paired florbetapir/PiB scans were downloaded from the GAAIN website (http://www.gaain.org/centiloid-project). SUVRs were calculated using the standard method from Klunk et al. [29] and the AAL template/gray matter-masked method used in this work. The Klunk SUVR method on level 1 ^11^C-Pib data (45 AD patients, 34 young control subjects) was replicated via a regression analysis with slope 0.994 and *R*^2^ = 0.99. A subsequent regression analysis of the level 2 paired florbetapir/^11^C-PiB allowed calculation of a single linear regression equation relating florbetapir AAL SUVR to Centiloid values. The linear transformations for this prespecified SUVR method are ^FBP^SUVR = 0.514 × ^PiB^SUVR + 0.749, *R*^2^ = 0.75; Centiloid = ^FBP^SUVR × 184.12 − 233.72 [29, 30], where ^FBP^SUVR and ^PiB^SUVR represent the AAL-calculated SUVR for florbetapir, and ^PiB^SUVR represents the Klunk SUVR method results for PiB, respectively.

Another important anchor for interpreting PET results is the threshold for amyloid positivity, which is the quantitative threshold that best discriminates pathologically verified absence of plaques or sparse plaques from moderate to frequent plaques. Navitsky et al. showed that a Centiloid value of 24 corresponds to the amyloid positivity threshold for florbetapir [30]. Independently, the Alzheimer’s Disease Neuroimaging Initiative (ADNI) PET core laboratory has established a positivity threshold of 1.11 by using a FreeSurfer method, whole cerebellum reference, and a corresponding Centiloid value of 24 [31]. Using the florbetapir data available on the GAAIN website (13 young controls, 33 elderly subjects) and calculating SUVR using both the ADNI FreeSurfer and AAL SUVR processing pipelines, it is found that the 1.11 ADNI positivity threshold corresponds to a 1.40 value using the AAL method with a cerebellar cortex reference (Additional file 4: Figure S2) [32]. Transformed into Centiloids, the 1.40 SUVR also corresponds to a 24-Centiloid threshold.

### Statistical analysis

The analysis population included all study participants receiving ≥ 1 OLE follow-up scan. All analyses presented here are exploratory in nature. Descriptive statistics were used to characterize patient demographics as well as change from baseline in PET Centiloid value and CDR-SB, ADAS-Cog 11, and MMSE scores. PET Centiloid value was also analyzed using a mixed model for repeated measures (MMRM), with visit, treatment group, and the interaction for treatment group by visit as independent variables. An unstructured covariance matrix was used to capture within-patient correlation. For descriptive group comparisons, the MMRM model was also applied to change from baseline in the clinical endpoint, with baseline value, group, and group-by-visit interaction as independent variables. Joint linear mixed effects (JLME) models were used to evaluate the association between the annual rate of change in PET and that in CDR-SB, ADAS-Cog 11, and MMSE scores while simultaneously controlling the impact of the PET baseline on the rate of change in the latter. For the ease of model convergence, PET, CDR-SB, ADAS-Cog 11, and MMSE scores were standardized to *z*-scores using the baseline mean and SD.

## Results

### Patients

A total of 379 patients were dosed in the SR and MR OLE trials; of these, 67 had scans at OLE week 52 and 39 had scans at OLE week 104 as of August 15, 2018 (Additional file 3: Figure S1). Patient characteristics at OLE baseline are summarized for OLE week 52 completers in Table 1. Amyloid loads at OLE baseline were 91, 80, and 50 Centiloids in the MR-DBP, MR-DBA, and SR groups, respectively (Table 1). Mean (SD) MMSE scores were 21.6 (4.5), 19.3 (5.0), and 18.8 (4.8), respectively, and ranged from 11 to 29. This indicates that although the original patient populations in the double-blind studies were prodromal/mild, some patients' health had declined by the time of OLE baseline, resulting in an overall mild/moderate patient population for this analysis. All OLE baseline characteristics of week 52 completers were broadly consistent with those of week 104 completers, except for mean baseline Centiloid value in the SR cohort, which was 70 in week 104 completers (not shown) but only 50 in week 52 completers.

**Table 1:** OLE baseline characteristics of patients with PET scan at OLE week 52

Characteristic	MR-DBP (*n* = 27)	MR-DBA (*n* = 21)	SR (*n* = 19)
Age at start of OLE, median (IQR), years	74 (69–77)	65 (59–79)	72 (70–78)
Female, *n* (%)	12 (44)	11 (52)	11 (58)
White, *n* (%)	27 (100)	20 (95)	19 (100)
*APOEε4* genotype, *n* (%)			
0	9 (33)	10 (48)	2 (11)
1	13 (48)	4 (19)	14 (74)
2	5 (19)	7 (33)	3 (16)
MMSE score at OLE baseline, mean (SD)	21.6 (4.5)	19.3 (5.0)	18.8 (4.8)
Centiloid at OLE baseline, mean (SD)	91.1 (46.8)	79.6 (51.6)	49.6 (52.8)
Duration on high dose, median (IQR), weeks^a^	35 (33–37)	42 (40–45)	25 (21–33)
Duration between baseline and week 52 PET scan, median (IQR), weeks	68 (60–82)	73 (64–88)	55 (54–58)
Patients with baseline PET scan below amyloid-β threshold, *n* (%)	0	3 (14)	7 (37)

^a^High dose: ≥ 6 doses (6 months) of 900 to 1200 mg gantenerumab

### Gantenerumab markedly reduced brain amyloid-β plaque levels

**Fig. 1:**
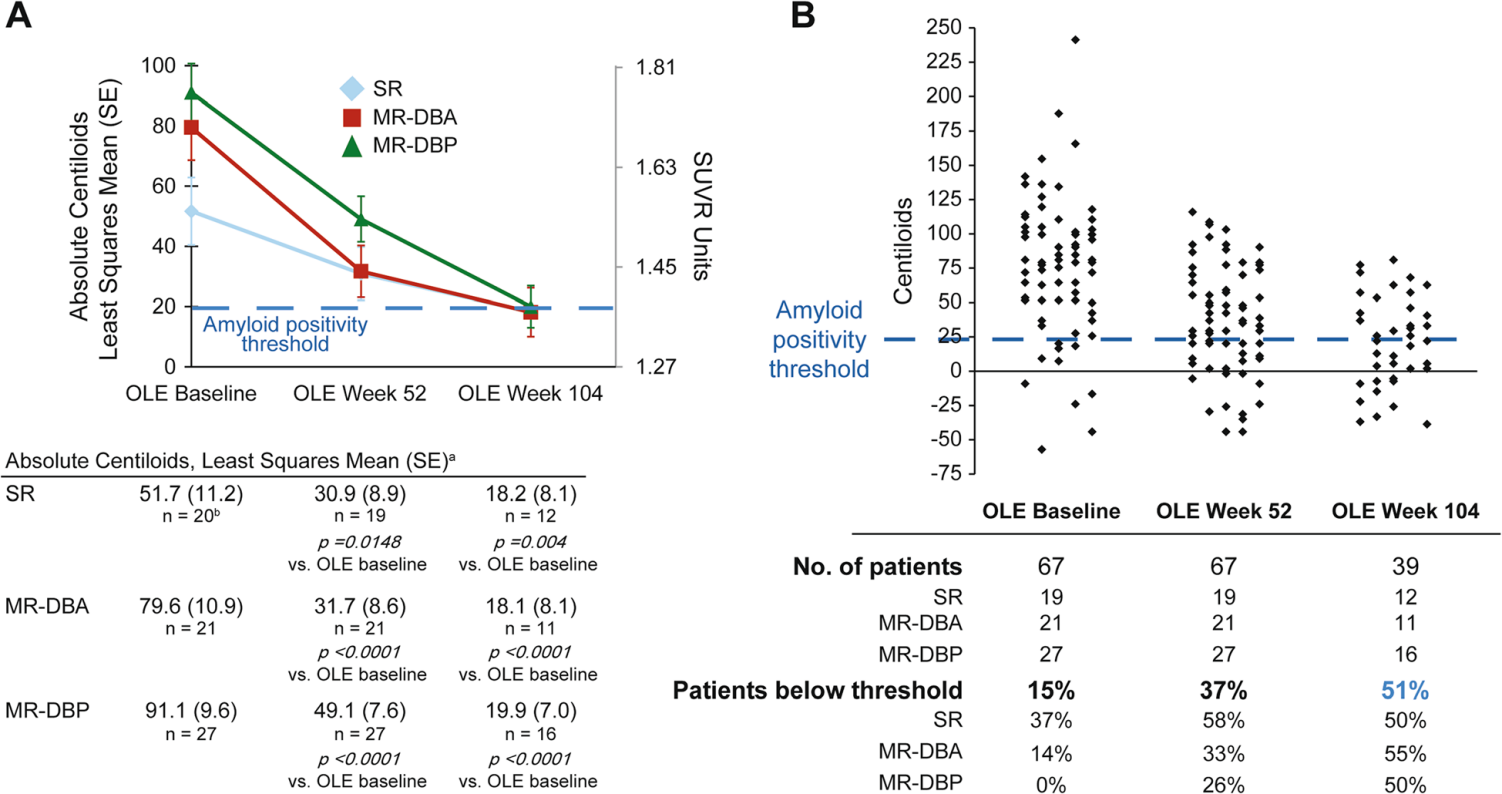
Marked and consistent reduction of amyloid load in patients receiving high-dose gantenerumab. **a** Marked reduction of amyloid-β plaques in patients receiving high-dose gantenerumab, and consistent reduction of amyloid-β plaques in all patient groups. **b** Reduction of amyloid-β plaque burden to below positivity threshold following high-dose gantenerumab. ^a^Analyzed using a mixed model for repeated measures

**Fig. 2:**
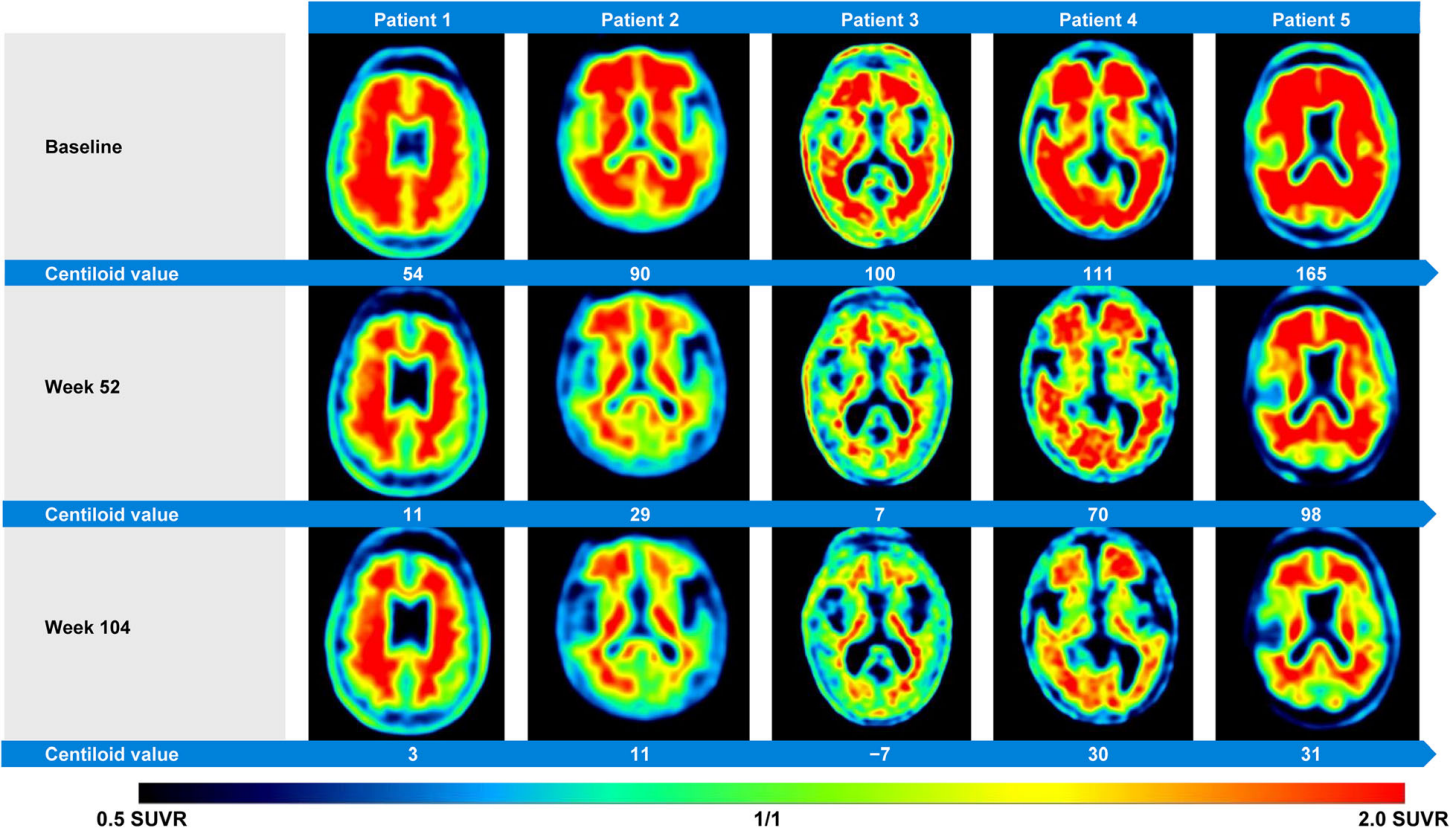
Amyloid-β plaque reduction with gantenerumab. Axial florbetapir brain PET images from five patients displaying reduction of amyloid-β plaques from OLE baseline to OLE week 52 and OLE week 104. Axial slices are at the level of the basal ganglia. PET images were obtained 50 min post-injection, SUVR data with the cerebellar cortex as the reference region

The reduction in amyloid PET was significant in all subgroups and at all time points. Mean (SE) PET Centiloid reductions from baseline were − 42 (7.1), − 48 (8.0), and − 21 (8.3) at week 52 and − 71 (7.7), − 61 (8.9), and − 34 (8.9) at week 104 in the MR-DBP, MR-DBA, and SR groups, respectively (Fig. 1); consistent results were observed for week 104 completers (Additional file 1: Table S1). Example images from five patients are shown in Fig. 2. Individually, all week 104 completers had reduced amyloid, with reductions ranging from 11 to 169 Centiloids, and 51% of patients had amyloid levels below the positivity threshold at week 104 (Fig. 1b). A strong correlation was seen between higher amyloid load at OLE baseline and greater amyloid reduction during the first year of gantenerumab treatment (Additional file 5: Figure S3). Large regional reductions were seen throughout the brain regions primarily involved in AD, with the greatest reductions observed in the anterior cingulate (Additional file 6: Figure S4). There were no observed effects of APOE status on PET reduction.

### Amyloid reduction and ARIA

In our analysis population of OLE PET week 52 substudy completers, 28 patients experienced ARIA-E, including 7 who reported symptoms. Global amyloid load at OLE baseline was not significantly different between the ARIA-E and non-ARIA-E groups (Table 2), and there were no significant differences between the ARIA-E and non-ARIA-E groups at either week 52 or 104; however, a directional trend toward slightly higher reductions in the ARIA-E group was observed (Table 2).

**Table 2:** Mean change in PET Centiloids from OLE baseline to OLE week 52 and OLE week 104 in patients with and without ≥ 1 ARIA-E event

	OLE week 52				OLE week 104			
Patients with ≥ 1 ARIA-E event	MR-DBP (*n* = 13)	MR-DBA (*n* = 8)	SR (*n* = 7)	Total (*n* = 28)	MR-DBP (*n* = 6)	MR-DBA (*n* = 3)	SR (*n* = 4)	Total (*n* = 13)
Centiloid composite ROI—mean cerebellum gray								
Baseline value, mean (SD)	90.47 (31.67)	68.01 (48.78)	67.19 (38.31)	78.23 (39.03)	82.35 (33.94)	63.94 (41.02)	78.83 (40.21)	77.02 (34.97)
Value at visit, mean (SD)	51.38 (23.42)	11.16 (38.04)	22.21 (28.82)	32.60 (33.73)	18.22 (21.77)	11.77 (50.63)	5.18 (24.58)	12.72 (28.46)
Change from baseline, mean (SD)	− 39.09 (30.90)	− 56.85 (29.52)	− 44.98 (33.43)	− 45.64 (30.93)	− 64.13 (20.28)	− 52.17 (28.66)	− 73.65 (33.89)	− 64.30 (25.72)
OLE week 52 change from baseline, mean (SD)	NA	NA	NA	NA	− 32.53 (31.50)	− 47.26 (12.93)	− 55.24 (40.53)	− 42.91 (31.01)
Patients without an ARIA-E event	MR-DBP (*n* = 14)	MR-DBA (*n* = 13)	SR (*n* = 12)	Total (*n* = 39)	MR-DBP (*n* = 10)	MR-DBA (*n* = 8)	SR (*n* = 8)	Total (*n* = 26)
Centiloid composite ROI—mean cerebellum gray								
Baseline value, mean (SD)	91.65 (58.77)	86.65 (53.87)	39.39 (58.71)	73.90 (60.36)	97.15 (68.72)	81.59 (66.35)	65.02 (42.53)	82.47 (60.19)
Value at visit, mean (SD)	46.93 (45.38)	44.30 (45.29)	32.95 (42.31)	41.75 (43.67)	25.34 (31.63)	18.53 (40.38)	34.64 (34.31)	26.10 (34.47)
Change from baseline, mean (SD)	− 44.72 (38.26)	− 42.35 (21.23)	− 6.45 (53.14)	− 32.15 (41.99)	− 71.81 (48.25)	− 63.06 (32.84)	− 30.38 (17.33)	− 56.37 (39.37)
OLE week 52 change from baseline, mean (SD)^b^	NA	NA	NA	NA	− 41.80 (38.85)	− 46.72 (20.26)	− 18.68 (16.79)	− 36.90 (29.92)

^a^*n* = 11 in SR and total *n* = 38 at OLE week 104

^b^*n* = 7 in SR and total *n* = 25 at OLE week 104

### Amyloid reduction and clinical results

**Fig. 3:**
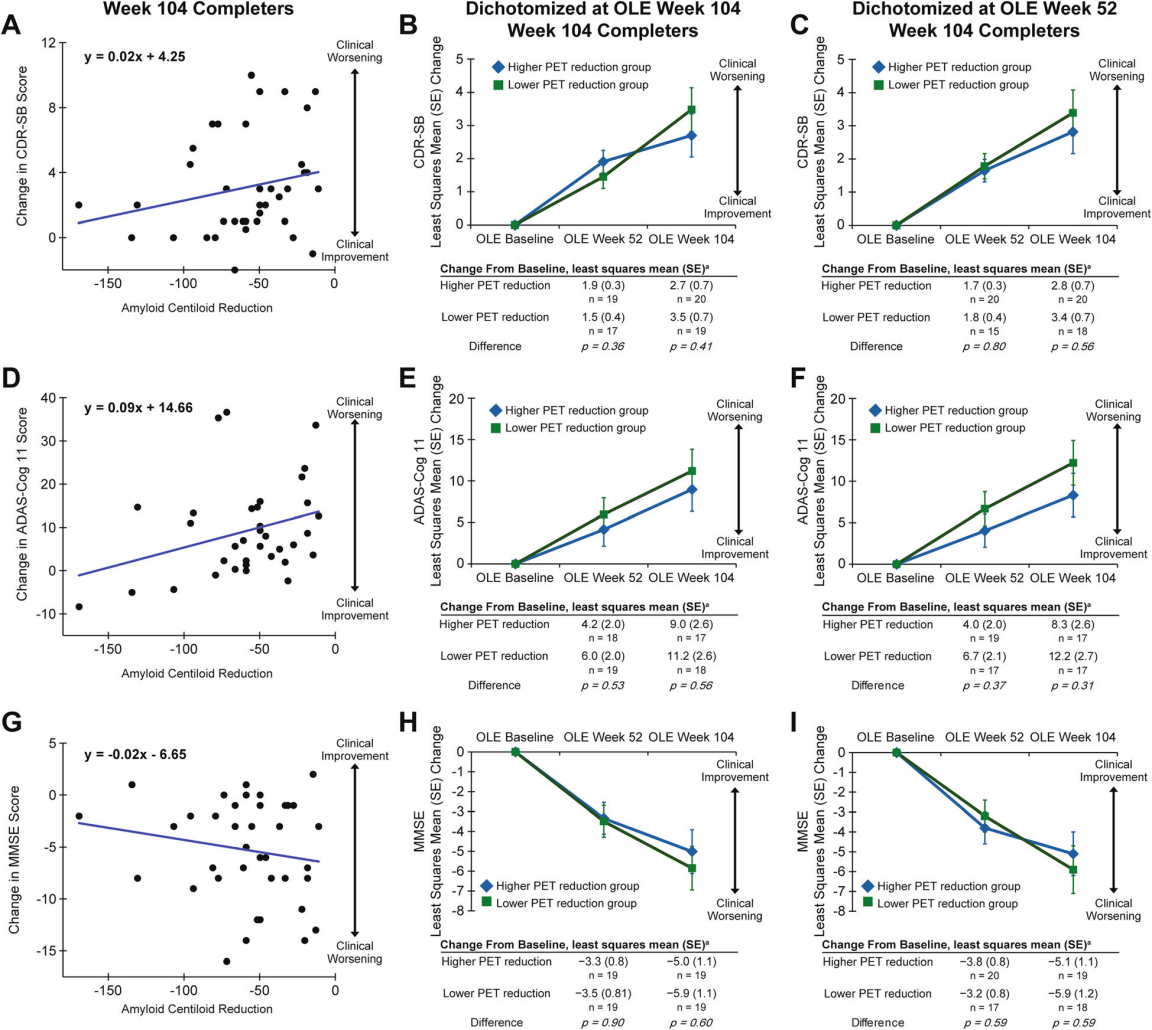
Association of PET and clinical results. **a**, **d**, **g** Regression analysis of CDR-SB, ADAS-Cog 11, and MMSE, respectively, shows directional slowing of clinical decline with increasing amyloid reduction. **b**, **e**, **h** Change in CDR-SB, ADAS-Cog 11, and MMSE for week 104 completers stratified by patients with high and low amyloid reduction at week 104 also shows directional slowing of clinical decline in the group with higher amyloid reduction. **c**, **f**, **i** Stratification of patients by amyloid reduction at the earlier week 52 time point shows increased clinical benefit in the high PET reduction group, suggesting a temporal lag between amyloid reduction and clinical benefit

This open-label, non-placebo-controlled trial was not designed to support analyses of clinical efficacy. However, exploratory analyses of clinical endpoints were performed. Baseline and follow-up CDR-SB, ADAS-Cog 11, and MMSE values are summarized in Table 3. Correlation analysis of change from baseline to week 104 among completers (patients with non-missing OLE week 104 PET data) suggested a directional trend for slower clinical decline with higher amyloid removal for all three endpoints (Fig. 3a, d, g).

**Table 3:** Mean change in clinical endpoints from OLE baseline to OLE week 52 and OLE week 104

	OLE week 52				OLE week 104			
MMSE total score	MR-DBP (*n* = 25)	MR-DBA (*n* = 21)	SR (*n* = 19)	Total (*n* = 64)	MR-DBP (*n* = 15)	MR-DBA (*n* = 11)	SR (*n* = 12)	Total (*n* = 38)
Baseline value, mean (SD)	21.56 (4.52)	19.29 (5.03)	18.84 (4.83)	20.07 (4.86)	22.31 (5.03)	21.55 (4.18)	19.58 (4.76)	21.26 (4.74)
Value at visit, mean (SD)	18.36 (5.08)	15.80 (5.99)	16.89 (6.60)	17.13 (5.85)	17.20 (7.16)	15.36 (7.26)	15.00 (8.26)	15.97 (7.41)
Change from baseline, mean (SD)	− 3.16 (2.81)	− 3.45 (3.63)	− 1.95 (3.34)	− 2.89 (3.25)	− 5.20 (4.39)	− 6.18 (5.34)	− 4.58 (5.26)	− 5.29 (4.87)
OLE week 52 change from baseline, mean (SD)	NA	NA	NA	NA	− 2.93 (2.52)	− 4.73 (3.82)	− 2.25 (3.62)	− 3.24 (3.36)
ADAS-Cog 11 score	MR-DBP (*n* = 24)	MR-DBA (*n* = 19)	SR (*n* = 19)	Total (*n* = 62)	MR-DBP (*n* = 14)	MR-DBA (*n* = 11)	SR (*n* = 10)	Total (*n* = 35)
Baseline value, mean (SD)	20.22 (8.21)	22.92 (10.83)	22.88 (7.92)	21.82 (9.00)	20.88 (10.20)	19.18 (10.03)	21.50 (9.21)	20.59 (9.64)
Value at visit, mean (SD)	24.39 (9.94)	28.72 (14.43)	29.61 (12.06)	27.32 (12.14)	28.48 (15.49)	31.03 (16.61)	26.63 (8.98)	28.75 (14.04)
Change from baseline, mean (SD)	4.28 (7.87)	5.82 (6.44)	6.74 (8.38)	5.51 (7.58)	8.17 (13.21)	11.85 (10.20)	7.87 (8.28)	9.24 (10.89)
OLE week 52 change from baseline, mean (SD)^a^	NA	NA	NA	NA	1.67 (7.09)	6.67 (7.78)	6.19 (9.27)	4.62 (8.17)
CDR-SB score	MR-DBP (*n* = 24)	MR-DBA (*n* = 19)	SR (*n* = 19)	Total (*n* = 62)	MR-DBP (*n* = 16)	MR-DBA (*n* = 11)	SR (*n* = 12)	Total (*n* = 39)
Baseline value, mean (SD)	5.04 (2.16)	5.14 (2.49)	5.84 (1.72)	5.30 (2.15)	4.28 (1.75)	4.36 (2.18)	5.25 (2.37)	4.60 (2.07)
Value at visit, mean (SD)	7.38 (3.02)	7.50 (4.21)	7.42 (2.84)	7.43 (3.32)	7.06 (3.73)	7.91 (5.75)	8.29 (4.29)	7.68 (4.45)
Change from baseline, mean (SD)	2.08 (2.37)	2.55 (2.05)	1.58 (1.73)	2.07 (2.10)	2.78 (2.85)	3.55 (3.90)	3.04 (2.89)	3.08 (3.12)
OLE week 52 change from baseline, mean (SD)^b^	NA	NA	NA	NA	1.39 (1.53)	1.55 (1.74)	2.00 (1.72)	1.64 (1.63)

^a^*n* = 12 in SR and total *n* = 37 at OLE week 104

^b^*n* = 14 in MR-DBP and total *n* = 36 at OLE week 104

“Higher” and “lower” PET amyloid reduction groups were defined based on the median PET Centiloid change from baseline to week 52 (Fig. 3c, f, i; median change threshold, − 34.99 Centiloids) or week 104 (Fig. 3b, e, h; median change threshold, − 55.23 Centiloids), among completers. The “higher” versus “lower” dichotomization was performed at two different time points to test the hypothesis that clinical benefit may follow amyloid reduction temporally [33]; in other words, higher amyloid reduction at week 52 may be associated with greater slowing of clinical decline at week 104, whereas this effect may be less pronounced when the time point for dichotomization of amyloid reduction and the clinical evaluation are contemporaneous. At week 104, point estimates for CDR-SB, ADAS-Cog 11, and MMSE showed numerically less decline in the higher vs lower PET amyloid reduction groups, defined at either OLE week 52 or 104. The differences between the higher and lower PET groups were non-significant with respect to change from baseline on all three clinical endpoints. When pooled together, results from the JLME analysis indicated a directional but not significant association between the annual reduction in PET and the annual change in CDR-SB, ADAS-Cog 11, and MMSE. For instance, an annual reduction of 0.54 SD in standardized *z*-score PET Centiloid would lead to a slowing of 0.55 SD in the standardized *z*-score ADAS-Cog increase. However, with the limited sample size and absence of a placebo arm, robust interpretation and conclusion from any such analysis is not possible.

## Discussion

This PET substudy of the MR and SR OLE studies investigated the effect of higher-dose gantenerumab on amyloid-β plaques over 104 weeks in patients with prodromal to moderate AD dementia. Reductions in PET values to below the amyloid-β positivity threshold were achieved in 37% of patients at week 52 and 51% at week 104, suggesting that amyloid-β plaques can be reduced to levels that would not support a neuropathological diagnosis of AD [34]. In addition, amyloid reductions were consistently observed across most patients, indicating that gantenerumab has the potential to reduce amyloid-β plaques as suggested by its proposed mechanism of action of preferential binding to amyloid-β aggregates and consequent plaque removal.

Compared with the PET mean (SD) change observed with the 225-mg dose during the SR 2-year double-blind period (− 0.09 [0.14] SUVR, − 16.6 [25.8] Centiloids) [23], reductions in this OLE substudy were markedly higher, with an overall reduction in all three cohorts at week 104 of 59.0 (35.3) Centiloids. The 64.8%, 77.3%, and 78.2% amyloid reductions in the SR, MR-P, and MR-NP groups, respectively, were well beyond the 2.4% ± 1.41% test-retest data reported for florbetapir [35]. Since patients in all three OLE cohorts achieved reductions in amyloid load at week 104 to near or below the positivity threshold, the lower mean reductions in the MR-DBA and SR groups compared with the MR-DBP group were most likely due to their lower baseline amyloid loads and not due to a difference in treatment effect among the three groups. The lower comparative reduction in the SR group at week 52 could also be explained by the shorter time on high dose compared with the MR-DBA and MR-DBP groups. Regional analyses indicate that amyloid reductions are fairly uniform throughout the regions of the brain known to have amyloid, indicating that sufficient blood-brain barrier penetration of gantenerumab has occurred to effect globally large amyloid reductions. Regarding PET amyloid reductions and ARIA-E, it was observed that while patients with ARIA-E may have a trend toward slightly higher amyloid reductions compared with patients without ARIA-E, both groups achieved very large amyloid reductions. Consequently, the gantenerumab data indicate that ARIA-E is not a prerequisite for large amyloid reductions. This result contrasts the data reported for bapineuzumab, in which there was a lack of change from baseline in the non-ARIA-E groups [36].

Although comparison of PET amyloid reductions with gantenerumab vs the effect seen with other anti-amyloid therapies is challenging [37], use of the Centiloid scale helps to better understand the current landscape of therapies with amyloid removal as a mechanism of action [29, 38]. To date, three other compounds have reported similar levels of amyloid reduction. The 10-mg/kg arm of the aducanumab PRIME phase 1b study had a baseline Centiloid value of 96 (SUVR, 1.44) and a reduction of 71 Centiloids at week 110 [33, 39]. The highest-dose group for BAN2401 had a 70-Centiloid reduction at 18 months from 74.5 Centiloids at baseline [40], while the LY3002813 highest-dose group showed a 68.8-Centiloid reduction from 111 Centiloids at baseline by week 24 [41]. The most representative gantenerumab OLE cohort for comparison is the previously untreated MR-DBP arm, which had a baseline load of 92 Centiloids and a reduction of 69 Centiloids by week 104. When comparing Centiloid reductions seen here to those in other studies, it is important to note that the rate of reduction appears to be related to baseline levels of amyloid as well as to drug effects.

Earlier anti-amyloid-β studies, including bapineuzumab and solanezumab, as well as BACE-1 studies, such as verubecestat, did not report amyloid values using Centiloid values, but all three of these showed no or modest amyloid-β reductions, < 5% with respect to baseline after treatment [42–44]. Other studies have reported amyloid decline in placebo groups [45], but the magnitude was far smaller than the amyloid reductions seen with the four high amyloid-removing monoclonal antibodies listed above.

There are several limitations to these analyses. The OLE studies reported here did not have any disease severity-related inclusion or exclusion criteria, and by the start of the OLE, some patients had progressed to a mild-moderate stage. The studies were neither randomized nor blinded and did not include a placebo arm; furthermore, the studies are still ongoing, with additional subjects expected to reach week 104. In addition, several measures could be considered to improve the robustness of the results. Results reported here are from static PET imaging, which may not account for certain drug effects (e.g., changes in blood flow or clearance). Dynamic imaging may produce slightly more robust results; however, the differences due to perfusion changes are likely to be small compared with the SUVR change seen here [46–48]. Our use of static PET is consistent with all previously reported findings on amyloid reduction with investigational AD therapies [33, 40, 43, 49, 50]. Brain atrophy progresses over time in AD, potentially even after amyloid aggregates are removed, and application of a partial volume correction (PVC) may be useful for interpreting our findings. PVC will be considered for future exploratory analysis. It should be noted, however, that evidence supporting PVC is mixed, with some reporting an improved signal [51] whereas others report greater noise [52, 53]. Furthermore, an analysis of cerebellar gray, pons, and white matter reference region SUVs compared to cortical target region SUVs (see Additional file 2: Table S2) shows that the SUVR change is primarily driven by reductions of signal in the target cortical regions, not the reference regions, and is not likely driven by spillover of cortical white matter activity.

A key step is to clarify how amyloid reductions, such as those reported here, correspond to any clinical benefit among patients with early symptomatic AD. Other randomized, parallel-group studies with aducanumab [39] and BAN2401 [40] have demonstrated PET amyloid signal reduction accompanied by indications of clinical efficacy in a similar patient population. In addition, a post hoc analysis of the SR trial demonstrated evidence of gantenerumab dose-dependent slowing of decline in some clinical efficacy scales [23]. Whereas two phase III studies of aducanumab were discontinued due to a low likelihood of reaching the primary endpoint [54], initially calling into question the relationship of amyloid reduction to clinical benefit, a subsequent analysis of the same studies reported evidence of an exposure-dependent clinical benefit associated with amyloid reduction [55]. (Full data from these phase III studies have not yet been published at the time of this writing.)

In this study, exploratory results showed a directional trend, whereby patients with higher PET amyloid reduction exhibited numerically less cognitive decline. Considering the limited sample size, heterogeneity among patients, and other potential confounders, including the observed relationship between baseline amyloid levels and amyloid reduction, caution must be used in the interpretation of clinical outcome data, particularly the comparison to previously published results. Nevertheless, these data do contribute to an overall body of evidence in the field that amyloid reduction is associated with clinical benefit among patients with early symptomatic AD, as outlined above. The hypothesis that higher-dose gantenerumab treatment contributes to clinical benefit in association with amyloid reduction is currently being tested in the ongoing GRADUATE phase III pivotal studies (NCT03444870, NCT03443973).

## Conclusions

We have shown that gantenerumab reduces amyloid-β plaques, one of the main pathological hallmarks of AD. These data and the favorable safety profile observed at these dose levels provide the rationale for further investigation of the clinical efficacy of gantenerumab, and two pivotal phase III trials in patients with early AD (GRADUATE 1 and 2) are currently ongoing [56, 57].

## Supplementary information

**Additional file 1: Table S1.** Reduction in Amyloid Load in Patients Receiving High-Dose Gantenerumab (Week 104 Completers). Findings in Week 104 completers show continued reduction in amyloid load across groups over 104 weeks, consistent with the overall group.

**Additional file 2: Table S2.** SUV Values for Week 104 Completers. Evaluation of cerebellar gray, pons, and white matter reference region SUVs compared with cortical target region SUVs show that SUVR change is primarily driven by changes in the target cortical regions.

**Additional file 3: Figure S1.** Study Design With Dosing Schedule and Patient Disposition. (A) Schematic representation of the MR and SR OLE study designs and dose-titration schedules. All patients in the OLE (including those previously on placebo) received gantenerumab subcutaneously every 4 weeks. Dose-titration schedules for uptitration to 1200 mg were assigned based on APOEε4 carrier status and last treatment dose during the double-blind phase. (B) Patient disposition. ^a^ Including 1 patient who missed their week 52 visit.

**Additional file 4: Figure S2.** Linear Regression of AAL SUVR (cerebellar gray reference) vs ADNI FreeSurfer (whole cerebellum reference). Linear regression of SUVR results computed on the same ADNI patients allows transformation of the previously published 1.11 amyloid-β positivity threshold to a value of 1.40 for the method here using a cerebellar cortex reference region. The 95% CIs (shaded area) were calculated using the bootstrap (quartile) method.

**Additional file 5: Figure S3.** Correlation Between Amyloid Load at OLE Baseline and Amyloid Change Over Time. Rate of amyloid reduction during the first year of gantenerumab treatment appears to be linked to baseline amyloid burden. Higher rates of amyloid reduction are seen with greater baseline burden.

**Additional file 6: Figure S4.** Regional Reductions in Amyloid Load**.** Amyloid reductions are seen in all regions known to be involved with amyloid pathology. Highest reductions are seen in the cingulate, frontal, and striatum areas. When adjusted for baseline amyloid burden, the caudate region shows the greatest regional reduction.

## Data Availability

The datasets analyzed during the current study are available from the corresponding author on reasonable request. Qualified researchers may request access to individual patient level data through the clinical study data request platform (www.clinicalstudydatarequest.com). Further details on Roche’s criteria for eligible studies are available here (https://clinicalstudydatarequest.com/Study-Sponsors/Study-Sponsors-Roche.aspx). For further details on Roche’s Global Policy on the Sharing of Clinical Information and how to request access to related clinical study documents, see here (https://www.roche.com/research_and_development/who_we_are_how_we_work/clinical_trials/our_commitment_to_data_sharing.htm).

## Abbreviations

AAL: Automated Anatomical Labelling
AD: Alzheimer’s disease
ADAS-Cog: Alzheimer’s Disease Assessment Scale cognitive subscale
ADNI: Alzheimer’s Disease Neuroimaging Initiative
APOEε4: Apolipoprotein E ε4
ARIA-E: Amyloid-related imaging abnormalities representative of vasogenic edema
CDR-SB: Clinical Dementia Rating Scale Sum of Boxes
LONI: Laboratory of Neuroimaging
MMSE: Mini-Mental State Examination
MR: Marguerite RoAD
MR-DBA: Marguerite RoAD double-blind active arm
MR-DBP: Marguerite RoAD double-blind placebo arm
NA: Not applicable
OLE: Open-label extension
PET: Positron emission tomography
q4w: Every 4 weeks
ROI: Region of interest
SC: Subcutaneous
SE: Standard error
SR: SCarlet RoAD
SUVR: Standard uptake value ratio
