# Supplementary material for: Gantenerumab reduces amyloid-β plaques in patients with prodromal to moderate Alzheimer’s disease: a PET substudy interim analysis
Source: Alzheimers Res Ther. 2019 Dec 12;11:101. doi: 10.1186/s13195-019-0559-z (PMC6909550; doi:10.1186/s13195-019-0559-z)
Supplement: Supplementary file 1 — Additional file 1: Table S1. Reduction in Amyloid Load in Patients Receiving High-Dose Gantenerumab (Week 104 Completers). Findings in Week 104 completers show continued reduction in amyloid load across groups over 104 weeks, consistent with the overall group. [file 13195_2019_559_MOESM1_ESM.docx]

# **Table S1**

|  | **Amyloid Burden, Least Squares Mean (SE)^a^ Centiloids** | | |
| --- | --- | --- | --- |
|  | **OLE Baseline** | **OLE Week 52** | **OLE Week 104** |
| **SR (n=12)** | 69.6 (15.4) | 38.0 (11.1)^b^ | 24.8 (9.7) |
| Change from baseline |  | –37 (6.4)  p=0.0009 | –50 (6.3)  p<0.0001 |
| **MR-DBA (n=11)** | 76.8 (16.0) | 29.9 (11.5) | 16.7 (10.1) |
| Change from baseline |  | –49 (6.5)  p<0.0001 | –62 (6.6)  p<0.0001 |
| **MR-DBP (n=16)** | 91.6 (13.3) | 53.3 (9.6) | 22.7 (8.4) |
| Change from baseline |  | –33 (5.4)  p<0.0001 | –64 (5.5)  p<0.0001 |

^a^Analyzed using a mixed model for repeated measures; ^b^n=11.
