# Supplementary material for: Gantenerumab reduces amyloid-β plaques in patients with prodromal to moderate Alzheimer’s disease: a PET substudy interim analysis
Source: Alzheimers Res Ther. 2019 Dec 12;11:101. doi: 10.1186/s13195-019-0559-z (PMC6909550; doi:10.1186/s13195-019-0559-z)
Supplement: Supplementary file 2 — Additional file 2: Table S2. SUV Values for Week 104 Completers. Evaluation of cerebellar gray, pons, and white matter reference region SUVs compared with cortical target region SUVs show that SUVR change is primarily driven by changes in the target cortical regions. [file 13195_2019_559_MOESM2_ESM.docx]

# **Table S2**

|  | **Cerebellar grey SUV** | **Pons SUV** | **White matter SUV** | **Cortical composite**  **SUV** | **Cortical composite SUVR** |
| --- | --- | --- | --- | --- | --- |
| Baseline | 0.795 | 1.407 | 1.522 | 1.343 | 1.706 |
| Week 52 | 0.839 | 1.479 | 1.577 | 1.248 | 1.493 |
| *Percent change from baseline* | *5.51* | *5.16* | *3.57* | *-7.09* | *-12.52* |
| Week 104 | 0.821 | 1.487 | 1.563 | 1.135 | 1.396 |
| *Percent change from baseline* | *3.23* | *5.68* | *2.68* | *-15.46* | *-18.19* |
