# Supplementary figures and images for: Gantenerumab reduces amyloid-β plaques in patients with prodromal to moderate Alzheimer’s disease: a PET substudy interim analysis
Source: Alzheimers Res Ther. 2019 Dec 12;11:101. doi: 10.1186/s13195-019-0559-z (PMC6909550; doi:10.1186/s13195-019-0559-z)

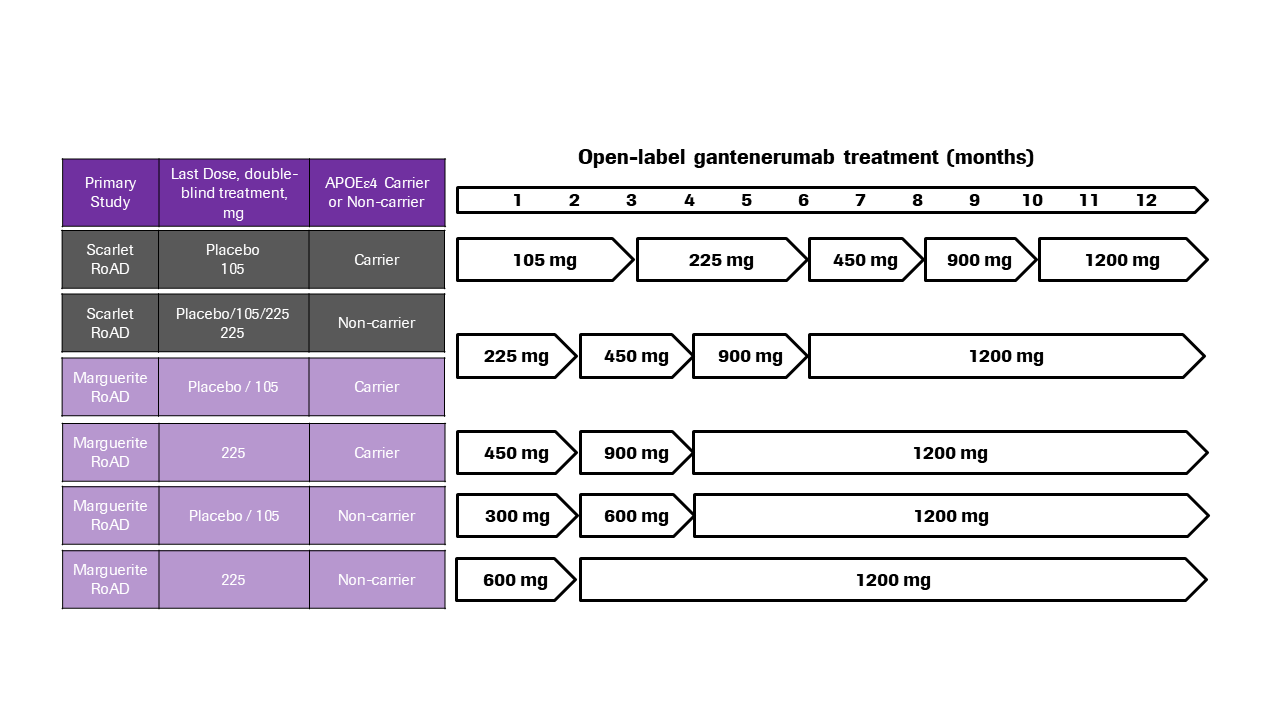

Supplement: Supplementary file 3 — Additional file 3: Figure S1. Study Design With Dosing Schedule and Patient Disposition. (A) Schematic representation of the MR and SR OLE study designs and dose-titration schedules. All patients in the OLE (including those previously on placebo) received gantenerumab subcutaneously every 4 weeks. Dose-titration schedules for uptitration to 1200 mg were assigned based on APOEε4 carrier status and last treatment dose during the double-blind phase. (B) Patient disposition. a Including 1 patient who missed their week 52 visit. [file 13195_2019_559_MOESM3_ESM.zip › Figure S1a.png]

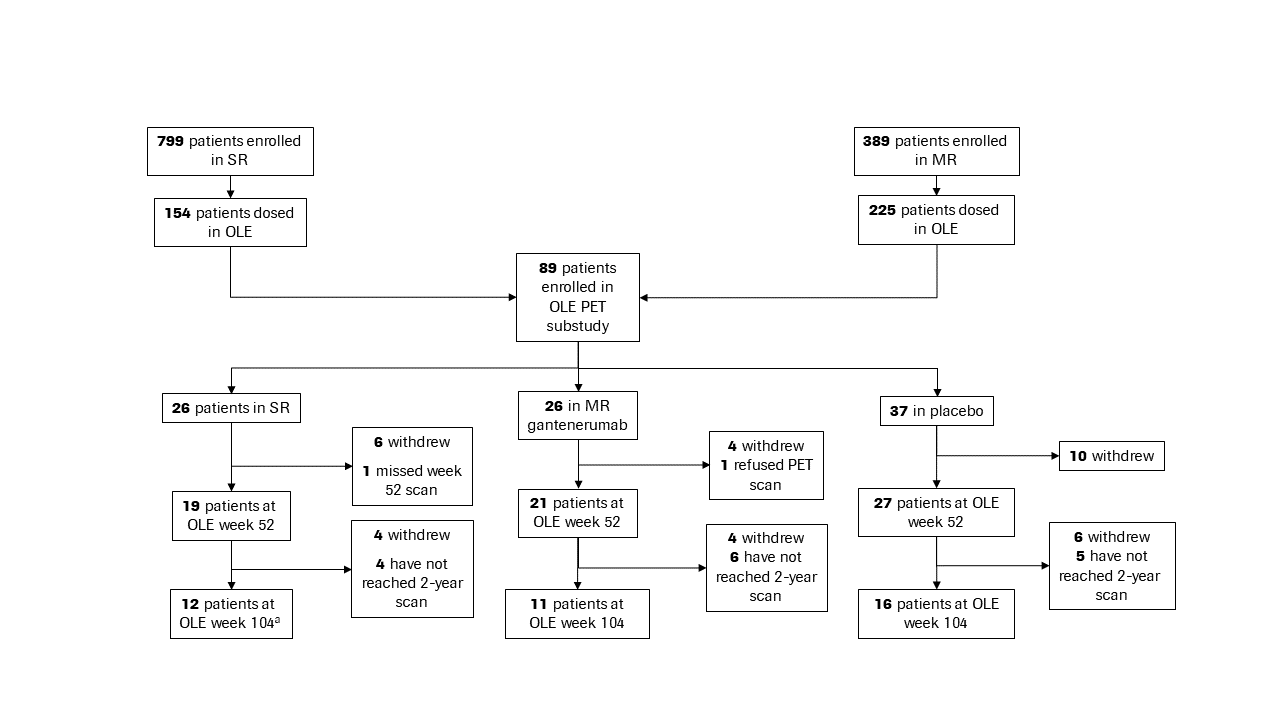

Supplement: Supplementary file 3 — Additional file 3: Figure S1. Study Design With Dosing Schedule and Patient Disposition. (A) Schematic representation of the MR and SR OLE study designs and dose-titration schedules. All patients in the OLE (including those previously on placebo) received gantenerumab subcutaneously every 4 weeks. Dose-titration schedules for uptitration to 1200 mg were assigned based on APOEε4 carrier status and last treatment dose during the double-blind phase. (B) Patient disposition. a Including 1 patient who missed their week 52 visit. [file 13195_2019_559_MOESM3_ESM.zip › Figure S1b.png]

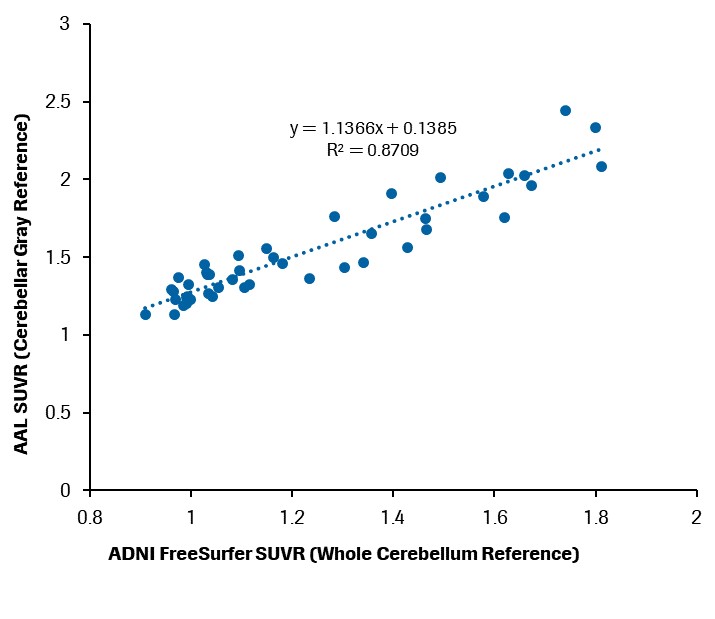

Supplement: Supplementary file 4 — Additional file 4: Figure S2. Linear Regression of AAL SUVR (cerebellar gray reference) vs ADNI FreeSurfer (whole cerebellum reference). Linear regression of SUVR results computed on the same ADNI patients allows transformation of the previously published 1.11 amyloid-β positivity threshold to a value of 1.40 for the method here using a cerebellar cortex reference region. The 95% CIs (shaded area) were calculated using the bootstrap (quartile) method. [file 13195_2019_559_MOESM4_ESM.jpg]

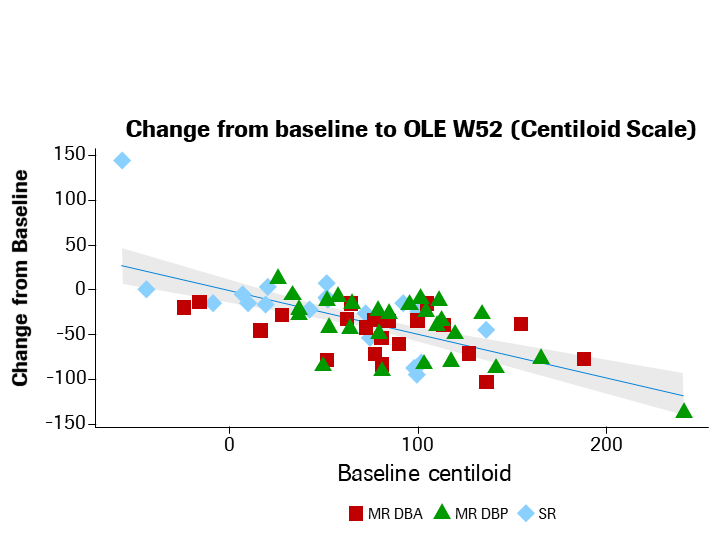

Supplement: Supplementary file 5 — Additional file 5: Figure S3. Correlation Between Amyloid Load at OLE Baseline and Amyloid Change Over Time. Rate of amyloid reduction during the first year of gantenerumab treatment appears to be linked to baseline amyloid burden. Higher rates of amyloid reduction are seen with greater baseline burden. [file 13195_2019_559_MOESM5_ESM.png]

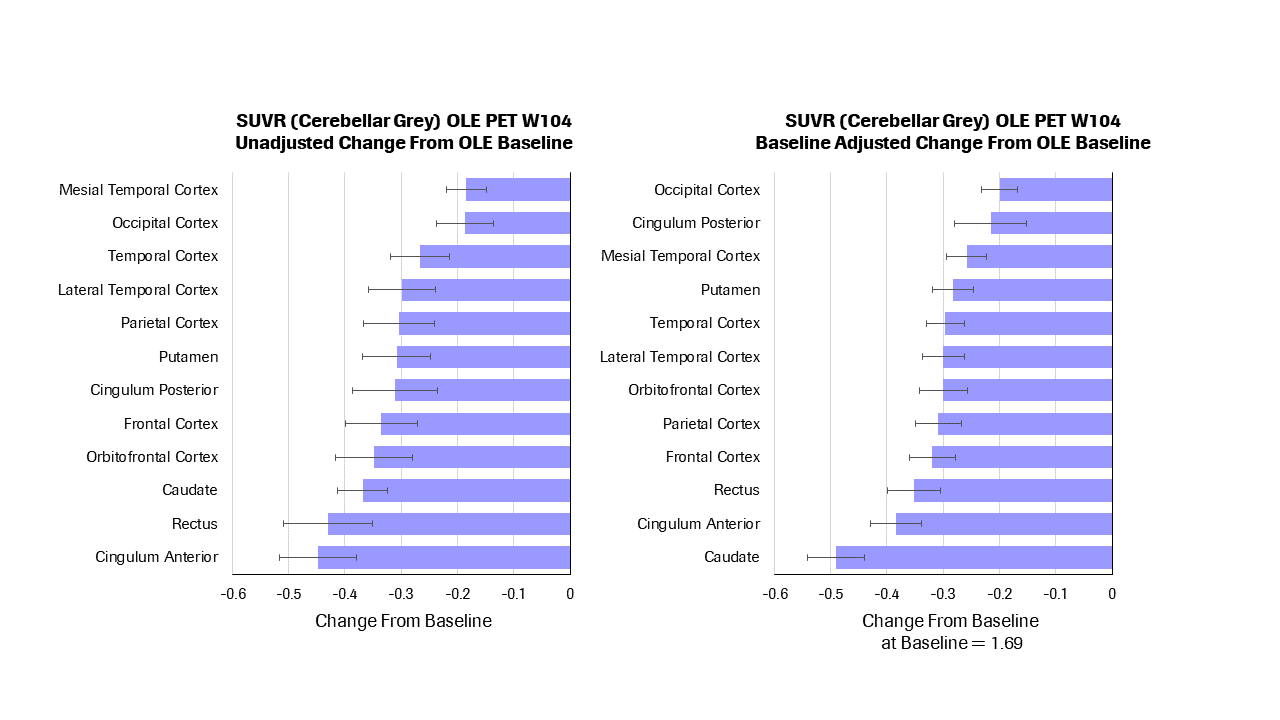

Supplement: Supplementary file 6 — Additional file 6: Figure S4. Regional Reductions in Amyloid Load. Amyloid reductions are seen in all regions known to be involved with amyloid pathology. Highest reductions are seen in the cingulate, frontal, and striatum areas. When adjusted for baseline amyloid burden, the caudate region shows the greatest regional reduction. [file 13195_2019_559_MOESM6_ESM.png]
